# Cardiovascular Involvement in Sepsis

**DOI:** 10.1155/2016/8584793

**Published:** 2016-05-18

**Authors:** Emanuela Turillazzi, Vittorio Fineschi, Cristian Palmiere, Consolato Sergi

**Affiliations:** ^1^Section of Legal Medicine, Department of Clinical and Experimental Medicine, University of Foggia, Ospedale Colonnello D'Avanzo, Via degli Aviatori 1, 71100 Foggia, Italy; ^2^Department of Anatomical, Histological, Forensic and Orthopedic Sciences, Sapienza University of Rome, Viale Regina Elena 336, 0016 Rome, Italy; ^3^University Center of Legal Medicine, Lausanne University Hospital, chemin de la Vulliette 4, 1000 Lausanne 25, Switzerland; ^4^Department of Laboratory Medicine and Pathology, University of Alberta, 8440 112 Street, Edmonton, AB, Canada; ^5^Stollery Children's Hospital, University of Alberta Hospital, 8440 112 Street, Edmonton, AB, Canada

This special issue wants to contribute to a better understanding of cardiac involvement in sepsis. Sepsis is a complex syndrome that has recently been defined as “life-threatening organ dysfunction due to a dysregulated host response to infection” [1, 2]. It should be considered a major public health problem since it affects millions of people worldwide each year, and it accounts for most deaths in critically ill patients. The presence of myocardial dysfunction in sepsis is associated with higher mortality.

A great attention has been dedicated to improving our knowledge and understanding of the intricate mechanisms underlying sepsis. However, data from the literature suggest the need to implement strategies to reliably measure sepsis morbidity and mortality. In fact, methods based on analyses of insurance claim data using sepsis-specific codes or separate codes for infection and organ dysfunction are unreliable in informing or measuring the effects of policy changes [3, 4], and the postmortem diagnosis of sepsis is often elusive since postmortem investigations lack certain pathognomonic macroscopic and histopathological findings [5, 6]. From a morphological and diagnostic point of view, the term “septic disease” has been created to describe the cardiac involvement in the syndrome. However, this definition, rather than describing a morphological finding, was instead referred to the clinical setting. Although in recent years the concept of septic cardiomyopathy has evolved and it involves pathological alterations of myocardial cells in response to the multiplicity of acting mechanism of damage, the importance of structural changes during sepsis is often overlooked. In patients with sepsis, death is usually the result of a progressive multiorgan dysfunction, overlooking the primary infection through the hyperinflammation. The cardiac involvement as fundamental part of septic multiorgan dysfunction syndrome has been discussed for a long time.

C. Pomara et al. explored the state of current knowledge on the molecular and biohumoral mechanisms of sepsis and correlate them with our ability at postmortem diagnosis. The authors highlight that a complete methodological approach, integrating clinical data by means of autopsy and histological and laboratory findings aiming to identify and demonstrate the host response to infectious insult, is mandatory. Such an approach would be likely to produce an accurate objective surveillance of deaths due to sepsis and improve our knowledge of the clinical-pathological correlation in sepsis, thus contributing to the evaluation of the effectiveness of therapies.

Sepsis is characterized by symptoms and manifestations of organ dysfunction that may lead to fatal outcome. Myocardial dysfunction in sepsis is mediated by a complex interplay among several factors that still remains incompletely understood [7]. Circulatory compromise and microcirculatory alterations may, in part, explain cardiac impairment in sepsis. However, in recent years increasing attention has been paid to the study of other possible pathways of myocardial dysfunction in sepsis. The effects of the host's immune-inflammatory response with particular focus on depressant molecules, complement molecules, cellular adhesion molecules, and altered intracellular energetic, dysregulated intracellular calcium fluxes have been called upon in the pathophysiology of myocardial depression in sepsis. Oxidative-nitrosative stress may contribute to cardiac dysfunction in sepsis, and mitochondria are one of the major sites for generation of reactive oxygen species and reactive nitrosative species as a detrimental side product of oxidative energy metabolism [8]. M. Neri et al. reviewed the evidence for a role of oxidative-nitrosative stress unbalance and mitochondria dysfunction in myocardial depression in sepsis. NADPH oxidase-derived reactive oxygen species, the role of mitochondria, and finally the involvement of nitric oxide and peroxynitrite as critical factors in mediating myocardial dysfunction in sepsis are discussed.

Measurement of biomarkers is a potential approach to early prediction of the risk of mortality in patients with sepsis. Over the years, a great amount of molecules has been proposed as potential biological markers [9]. However, only 20% of these biomarkers have been assessed specifically in appropriate studies for use in the diagnosis of sepsis [10]. To date an ideal clinical and postmortem marker of sepsis does not exist. Proadrenomedullin and copeptin are peptides cosynthesized together with adrenomedullin and vasopressin in endothelial cells and pituitary gland, respectively. These peptides are increased during sepsis. They have vasoactive, immune modulating, and metabolic properties. It was recently reported that adrenomedullin plays a central role in initiating the hyperdynamic response during the early stages of sepsis and was a useful predictor for development of severe sepsis and septic shock [11, 12]. W. Hu et al. discuss the prognostic value of adrenomedullin and atrial and brain natriuretic peptides in uroseptic patients induced by ureteroscopy. In their research article the authors suggest that the prognostic value of adrenomedullin is superior to atrial and brain natriuretic peptides and that all these molecules are robust independent predictors of in-hospital death in uroseptic patients. They conclude that adrenomedullin and atrial and brain natriuretic peptides may participate in initiating the hyperdynamic response during the early stages of sepsis in uroseptic patients and that these biomarkers could be considered strong predictors of adverse outcome in patients with urosepsis.

Finally, two articles of the special issue discuss the potential link existing between inflammatory status and cardiometabolic disorders. It has been well established that inflammation is also able to affect lipoprotein metabolism. However, the precise mechanisms pathophysiologically linking metabolic dysfunction and inflammation are still under investigation. The biological basis for these associations includes both systemic and local tissue effects.

Metabolic Syndrome (MetS) is a constellation of diseases that include obesity, diabetes, hypertension, dyslipidemia, hypertriglyceridemia, and hypercholesterolemia. These metabolic derangements trigger a persistent inflammatory cascade, whit recruitment of immune cells to the site of injury, and subsequent expression of cytokines and chemokines that amplify the inflammatory response [13]. A common link between inflammation and MetS has been suggested. In cardiometabolic disorders a high serum lipopolysaccharides activity has been demonstrated, thus suggesting a potential role of bacterial infections and immune response in their etiology [14]. One article discussed the serum adiponectin levels and the homeostasis model assessment-insulin resistance (HOMA-IR) that are reportedly associated with MetS. Y.-S. Ding et al. concluded that the adiponectin to HOMA-IR ratio (A/H) may be a better diagnostic marker for MetS than either HOMA-IR or adiponectin alone, and it may serve as an important biomarker to determine an increased risk for MetS in healthy middle-aged population. Y. Zhang et al. investigated the relationship between inflammatory markers and the atherogenic lipoprotein subfractions, and they hypothesized presence of heterogeneity in the relationship of systemic inflammatory markers with atherogenic lipoprotein subfractions, which would aid our understanding of their interplay in the pathogenesis of atherosclerotic disease.

This special issue is dedicated to the cardiovascular involvement in sepsis. The high mortality associated with sepsis makes a thorough knowledge of its underlying mechanisms and its pathophysiological connections with the cardiometabolic disorders important.
